# Selection of reference genes for quantitative real-time PCR normalization in the plant pathogen *Puccinia helianthi* Schw.

**DOI:** 10.1186/s12870-019-1629-x

**Published:** 2019-01-11

**Authors:** Yang Song, Yan Wang, Dandan Guo, Lan Jing

**Affiliations:** College of Agronomy, Inner Mongolia Agricultural University, Hohhot, 010019 China

**Keywords:** Reference gene, *Puccinia helianthi* Schw., Sunflower, RT-qPCR, Algorithms

## Abstract

**Background:**

Real-time RT-PCR has become a common and robust technique to detect and quantify low-abundance mRNA expression and is a prefered tool when examining fungal gene expression in infected host tissues. However, correct evaluation of gene expression data requires accurate and reliable normalization against a reference transcript. Thus, the identification of reference genes with stable expression during different conditions is of paramount importance. Here, we present a study where in vitro and *in planta* experiments were used to validate the expression stability of reference gene candidates of *Puccinia helianthi* Schw**.**, an obligate pathogen that causes rust in sunflower (*Helianthus annuus*).

**Results:**

Eleven reference genes of *P. helianthi* were validated at different growth stages. Excel-based software geNorm, BestKeeper and NormFinder were used to evaluate the reference gene transcript stabilities. Of eleven reference gene candidates tested, three were stably expressed in urediniospores, germinating growth stage and *in planta*. Two of these genes (*UBC*, *EF2*), encoding ubiquitin-conjugating enzyme and elongation factor 2, proved to be the most stable set of reference genes under the experimental conditions used.

**Conclusion:**

We found that *UBC* and *EF2* are suitable candidates for for the standardization of gene expression studies in the plant pathogen *P. helianthi* and potentially other related pathogens.

## Background

Sunflower rust caused by the Basidiomycete *Puccinia helianthi* is one of the most destructive disease in major sunflower producing areas worldwide. It is common and widespread in China, occurs annually on cultivated sunflower (*Helianthus annuus* L.) and naturalized wild annual species. This obligate biotrophic fungus has several developmental stages varying in form and function, all within the sunflower host. The most effective control measure is the use of resistant varieties and hybrids. However, factors that regulate the pathogenesis of *P. helianthi* are unknown. Therefore, the study of expression patterns of key genes involved in an interaction between *P. helianthi* and sunflower at the molecular level would help in the breeding of resistant sunflower cultivars.

Biological techniques for detecting gene expression levels include: semi-RT-PCR, Northern blot, RNase protection assays, gene chips, RNA sequencing and quantitative real-time polymerase chain reaction (qRT-PCR). qRT-PCR is regarded as the most reliable technique for conducting simultaneous measurements of the relative levels of gene transcripts in many different samples because of its efficiency and sensitivity [1, 2]. Compared to conventional methods, qRT-PCR is the only method available for detecting low copy number mRNA of selected genes [3]. However, the accuracy of the results obtained by this method depends on accurate target transcript normalization using suitable reference genes, which can control potential experimental errors [4].

qRT-PCR has been used for pathogen detection like bacteria, viruses and fungi and gene expression analyses in plant tissues and soil [5, 6]. In many fungi, where the possibility to engineer the genomes is limited because of lack of regeneration and transformation protocols, qRT-PCR has become a frequent first choice for gene expression studies. The general molecular strategy to study fungal biology is to evaluate gene expression levels in an attempt to correlate transcript levels of specific genes to fungal response and adaptation to environmental conditions [7–9]. Furthermore, *P. helianthi* transcriptome data provide a powerful tool for putative gene selection and subsequent primer design for qRT-PCR experiments. However, use of the qRT-PCR technique must rely on a systematic process of reference gene evaluation to be included in the normalization step.

Recently there has been an increase in the number of reference gene validation studies in plant fungal pathogens such as *Puccinia* sp. in wheat rusts [10], *Fusarium* sp. in wheat head blight [11], *Aspergillus* sp. in black mould [12] and *Magnaporthe* sp. in rice blast [13]. Studies have shown that expression stability of a reference gene varies between species, and that expression could also vary across tissue type, developmental stages and experimental situations [14–17]. Therefore it is essential to screen a proper constitutively expressed control gene in different growth stages to be used as the internal controls for real time PCR.

Because of the intrinsic difficulties in genome manipulation in *P. helianthi*, we focused on gene expression analysis using qRT-PCR. The identification of valid endogenous control genes for gene expression normalization would facilitate further studies on *P. helianthi* development and interaction with the host at the transcript level. We identified the stability of 11 reference genes that were selected based on the transcriptome datasets of *P. helianthi*. Excel-based software were used to evaluate the stability of the candidate reference genes. This provided an extensive validation of 11 putative reference genes that can be applied to future gene expression study related to this fungus.

## Methods

### Rust pathogen isolates and sunflower

Races 330 and 737 of *Puccinia helianthi*, and sunflower (*Helianthus annuus* L.) varieties Heidapian (confectionery open pollinated variety) and 7350 (confectionery inbred line) were chosen. Heidapian showed susceptibility to race 330 and 7350 was susceptible to race 737, both consisted compatible combinations.

### Inoculation

The stored urediniospores of race 330 and 737 were inoculated on two week old susceptible plants to propagate fresh urediniospores. After 15 days, fresh urediniospores of each race were collected from rusted leaves by flicking leaves against parchment paper, and a portion of the spores were stored at − 80 °C, while the rest were used to inoculate sunflower plants.

Seeds of the sunflower varieties Heidapian and 7350 were surface disinfected in 1% sodium hypochlorite and planted at five seeds per pot (14 × 11 cm) containing pasteurized soil (total 5 pots for each variety). Races were smear-inoculated separately onto V2 stage seedlings with the concentration of 1 × 10^6^ spores·mL^− 1^. The inoculated seedlings were incubated at 20 ± 1 °C in a dew chamber (relative humidity 90–100%) for 24 h in the dark before shifting to a greenhouse at 20 ± 1 °C with a 16 h photoperiod (relative humidity 60–70%). After 5, 10 and 15 days, three inoculated leaves were respectively sampled, and stored in liquid nitrogen at − 86 °C. Healthy sunflower leaves of the two varieties were also sampled to test primer specificity.

### Sample preparation

Five treatments (Tr) were set up for each race and a variety combination. One group included urediniospores of race 330 (Tr1), 8 h geminated spores with germ tubes of race 330 (Tr2), 5 d inoculated Heidapian leaf with 330 (Tr3), 10 d inoculated Heidapian leaf with 330 (Tr4), and 15 d inoculated Heidapian leaf with 330 (Tr5). The other group included urediniospores of race 737 (Tr6), 8 h geminated spores with germ tubes of race 737 (Tr7), 5 d inoculated 7350 leaf with 737 (Tr8), 10 d inoculated 7350 leaf with 737 (Tr9), and 15 d inoculated 7350 leaf with 737 (Tr10).

In order to obtain germinated spores, 600 mL sterilized water containing fresh urediniospores upto the final concentration of 20 mg urediniospores·L^− 1^ was added into a 90 × 45 cm plate. After 8 h incubation at 15 °C in the dark, germinated urediniospores were collected, dried with blotting paper after high speed centrifugation, then immediately submerged in liquid nitrogen, and stored at − 80 °C. All treatments were repeated three times.

### RNA isolation from rust isolates and infected plant material

Total RNA was isolated from different samples including urediniospores, germinated spores of each race, and inoculated sunflower leaves sampled at 5, 10 and 15 days post inoculation (330/Heidapian and 737/7350) using the RNeasy Plant Mini Kit (QIAGEN Cat. No. 74904) following the manufacturer’s instructions. The concentration and purity of isolated RNA was measured with a UV spectrophotometer (NanoDrop ND-1000). The mean ratio value of A260/280 for all RNA samples was 2.0–2.1, A_260/230_ ≈ 2.0 reflecting high purity and protein absence. RNA integrity was verified by performing 1.5% agarose gel electrophoresis. To guarantee the quality necessary for expression analysis all samples presented a 28S/18S rRNA ratio ≥ 1.7.

### DNAse treatment and cDNA synthesis

Total RNA (25 μg) from each sample was treated with RQ1 RNase-Free DNase (Promega) in the presence of RNase inhibitor (Recombinant RNasin® RNase Inhibitor, Promega), following the manufacturer’s instructions. Treated RNA was reverse transcribed using the M-MLV reverse transcriptase (Cat. No. 28025–013; Invitrogen) in 20 μL of final volume, according to the manufacturer’s recommendations. The cDNA obtained after the total RNA amplification was separated on a 1.5% agarose gel to verify the integrity and product size. The cDNA quantification was performed with a UV spectrophotometer (NanoDrop ND-1000).

### Quantitative real-time RT-PCR

Eleven housekeeping genes were chosen as reference genes based on the *P. helianthi* transcriptome data, including β-actin (*ACTB*), elongation factor 1 (*EF1*), elongation factor 2 (*EF2*), elongation factor 3 (*EF3*), ribosomal protein S24 (*RPS24*), ribosomal protein S5 (*RPS5*), α-tubulin (*TUBA*), β-tubulin (*TUBB*), ubiquitin-conjugating enzyme (*UBC*), E2 ubiquitin-conjugating enzyme (*UBCE2*), polyubiquitin (*UBQ*). The gene sequences are stored in GenBank (Table 1).

**Table 1:** Selected housekeeping genes for expression analysis

Gene name	Gene symbol	Accession number	Function
Beta-actin	*ACTB*	KU355755	Cytoskeletal structural protein
Elongation factor 1	*EF1*	KU355750	Facilitate translational elongation
Elongation factor 2	*EF2*	KU355753	Facilitate translational elongation
Elongation factor 3	*EF3*	KU355748	Facilitate translational elongation
Ribosomal protein S24	*RPS24*	KU355746	Catalyze protein synthesis
Ribosomal protein S5	*RPS5*	KU355749	Catalyze protein synthesis
Alpha -tubulin	*TUBA*	KU355754	Cytoskeletal structural protein
Beta -tubulin	*TUBB*	KU355745	Cytoskeletal structural protein
Ubiquitin-conjugating enzyme	*UBC*	KU355747	Ubiquitination reaction
E2 ubiquitin-conjugating enzyme	*UBCE2*	KU355752	Ubiquitination reaction
Polyubiquitin	*UBQ*	KU355751	Ubiquitination reaction

All primers employed were designed using Primer 5 software (http://www.premierbiosoft.com/primerdesign/) and synthesized by Invitrogen. The criteria for primer design were as follows: primer lengths of 20–22 bp, GC contents of 45–55%, melting temperature (Tm) in a range of 55–60 °C and amplicon lengths of 100–150 bp.

In order to verify their specificity for *P. helianthi*, we used common PCR to test amplication in sunflower (cDNA were from varieties Heidapian and 7350). Only primers of housekeeping genes which had no amplification for sunflower were retained for qRT-PCR (Table 2).

**Table 2:** The primer sequences and amplification efficiency of candidate reference genes

Gene symbol	Forward primer(5′~ 3′)	Reverse primer(5′~ 3′)	Tm (°C)	Amplicon size (bp)	Application efficiency (%)
*ACTB*	TGGTTTGGAAGCATCCGGTA	TGTCGGAGATGCCCGAATAC	56	134	94.7
*EF1*	CCTTCACCCCTCTGTACTGC	CCACATGTTTGGGCGGATTG	56	113	86.5
*EF2*	TGGTCATCCGAACGACAAGG	GCCTAGGATGCCGTAAGGAC	57	130	99.0
*EF3*	ATGTCTGCCGATGAAGCCAA	AAGTCGGACGAAGGCTTGAG	57	132	98.1
*RPS24*	TGCTTTCGCTGGAGTAGACG	CAGTTCATCCCGGTCCTACC	56	107	97.8
*RPS5*	GGCAAGTAGCGTGCGAATTT	GACACATGGGTGGAGACGAA	56	149	98.9
*TUBA*	GACACCAAGACAGACACAGGT	TCTTTGCTTTCAACCACACCC	56	102	99.5
*TUBB*	GCCAGAAAGAAGTTGCGACC	TTCTCCAGTTTGACTGGCCG	56	141	101.7
*UBC*	GTGGTGACCAAAGACTGAGACA	CGACAGTCAGCCAACCTACC	56	119	95.0
*UBCE2*	GTTCTAGCGGGGTTTGTGGA	TTGCAGAAAAGCCCATTCGC	57	149	101.5
*UBQ*	GGCAGAGGACCACAAAGTCA	GGGATGTACACCTGTGAGCC	56	105	93.1

*ACTB* β-actin, *EF1* Elongation factor 1, *EF2* Elongation factor 2, *EF3* Elongation factor 3, *RPS24* Ribosomal protein S24, *RPS5* Ribosomal protein S5, *TUBA* α-tubulin, *TUBB* β-tubulin, *UBC* Ubiquitin conjugating enzyme, *UBCE2* E2 ubiquitin-conjugating enzyme, *UBQ* Polyubiquitin

The qRT-PCR was performed in 96 well plates with 20 μL total reaction volume for each sample/gene as follows: 10 μL Power SYBR® Green PCR Master Mix (2×) (PE Applied Biosystems, USA), 0.5 μL forward primer (final concentration 2.5 μM), 0.5 μL reverse primer (final concentration 2.5 μM), 1 μL (10 ng) of diluted cDNA, and nuclease-free water 8 μL. The following qRT-PCR program was used on a 7900 HT Fast RealTime PCR system (Applied Biosystems, USA): 15 s denaturation at 95 °C, 40 amplification cycles of 10 s at 95 °C, 1 min annealing 60 °C, and extension 1 min at 60 °C. The melting curve analysis was performed from 60 °C to 95 °C to verify primer specificity. Three biological replicates for each sample together with two technical replicates for each well were performed. Two negative controls in which the cDNA was replaced with nuclease free water were also included for each primer pair. Realtime data were analyzed using the ABI PRISM 7900 HT Software Tool (Applied Biosystems). Amplification efficiency of each primer pair was evaluated by the standard curve method using serial dilutions of pooled cDNA and the Relative Expression Software Tool (REST) was used for calculations [18]. All primer pairs presented amplification efficiency between primer efficiency 80–110%, r^2^ value 0.98–0.99 meeting the requirement for qRT-PCR. Primers were tested for non-specific product/s by amplicon separation on 2% (*w*/*v*) agarose gel electrophoresis at the end of a qRT-PCR run.

### Statistical data analysis

We applied three mathematical algorithms, geNorm [19], NormFinder [20] and BestKeeper [21] for evaluation of expression stability of the candidate reference genes.

Using geNorm to assess the best reference genes in *P. helianthi*, the CT values were transformed to relative expression levels and then calculated according to the manual. This program determines the pairwise variation of a reference gene with all other tested candidate genes and defines the gene expression stability measure M as the average pairwise variation between a particular gene and all other control genes. Genes with the most stable expression have the lowest M values. Stepwise exclusion of the gene with the highest M value ranks the tested genes according to their expression stability, resulting in a combination of two most stable genes left. The pairwise variation (Vn/n + 1) is calculated between the normalization factors to determine the optimal number of reference genes needed for normalization. The cut-off threshold value was set to V = 0.15, below which the inclusion of an additional reference gene is not required, as suggested by Vandesompele et al. [19].

The results of the stability rankings obtained from three algorithms were integrated, generating a comprehensive ranking according to the geometric mean of corresponding rankings. CT values were converted into raw relative quantities considering the PCR efficiency.

## Results

### Primer specificity and efficiency check

Target fragments were obtained from the rust cDNA amplification with all tested primers. However, the *ACTB* primers also gave a weak signal in the non-infected leaves (results not shown). None of the other primer pairs gave amplicons in the non-infected leaves, confirming the absence of *P. helianthi* in these controls. Therefore this pair of primers cannot be used as reference gene for Real time PCR to study rust gene expression.

Melting curves of amplified products with the remaining ten pairs of primers showed that a single amplification product of the expected size was obtained. There was no amplification, or high CT values (> 35 CT), for both non-template controls, minus reverse transcriptase (−RT) and the host-only control. This showed that the reagents were free from contamination as there was no amplification of gDNA and no non-specific amplification of plant cDNA. Calculation of primer efficiencies using five-fold dilution of pooled cDNA for all ten housekeeping gene primers gave r^2^ > 0.99 and 87–102% efficiency (E) values (Table 2).

### Expression levels of candidate reference genes

**Fig. 1:**
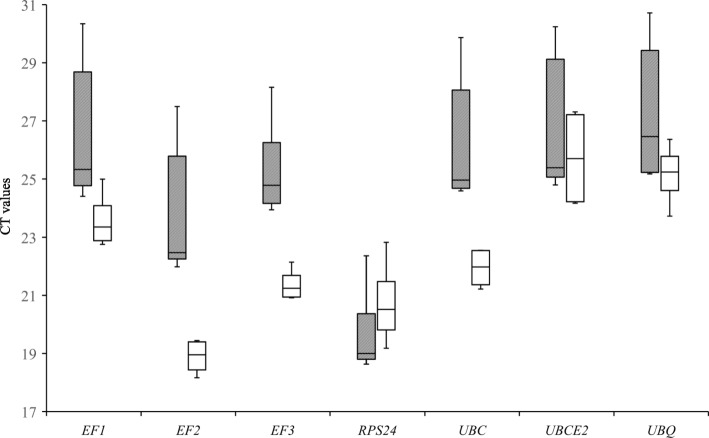
Expression levels of candidate housekeeping genes in pathogen and infected leaves. Boxes represent lower and upper quartiles of cycle thresholds range with medians indicated, whisker caps represent maximum and minimum values. Hatched boxes correspond to pathogen samples (urediniospores and germinated spores) and white boxes to infected leaves samples (5, 10 and 15 d post inoculation)

For each candidate gene, the CT value can be used to compare gene expression levels. The coefficient of variation of CT values reflects the stability of gene expression, wherein higher CT values correspond to lower gene expression levels and vice versa. Because expression levels of *RPS5*, *TUBA*, and *TUBB* in urediniospores, germinated spores, and inoculated leaves were very low (results not shown), three internal candidate genes (*RPS5*, *TUBA*, and *TUBB*) were excluded from subsequent analyses. Expression level of the remaining seven reference genes in different samples showed some variability. Among these, *EF2* and *RPS24* had the highest expression, while *UBCE2* and *UBQ* had lower expression (Fig. 1). These candidate genes lacked in regularity and stability of gene expression in different samples.

**Fig. 2:**
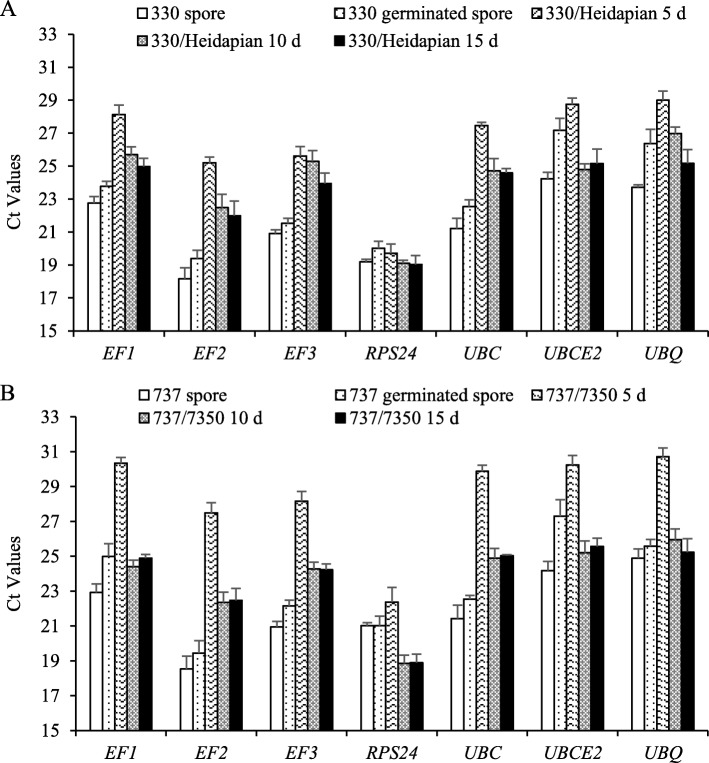
Expression levels of seven housekeeping genes throughout the different development of *P. helianthi*. **a** Expression levels of seven genes at different stages of race 330 (interacting with variety Heidapian). **b** Expression levels of seven genes at different stages of race 737 (interacting with variety 7350). Genes were normalized to different reference genes. Error bars show the standard error calculated from three biological replicates

The seven remaining reference gene candidates (*EF1*, *EF2*, *EF3*, *RPS24*, *UBC*, *UBCE2*, and *UBQ*) were subjected to further analyses. The expression in pure pathogen and *in planta* could not be directly compared, as the amount of starting concentration of fungus or fungal RNA could not be equalized, but the expression could be compared with respect to variation between different genes. RT-PCR analysis of RNA collected from 5, 10, and 15 day old leaves after infection showed that six of the candidate reference genes had similar expression levels in all samples, whereas the *RPS24* gene showed a clear up-regulation of mRNA expression (lower CT value) in infected plant material compared with urediniospores and germinated spores, and relative to the other reference gene candidates (Fig. 2). We thus discarded this gene as candidate housekeeping gene. To obtain a statistical evaluation of the candidates, we performed another statistical test to rank the remaining genes according to expression stability. We chose 10 and 15 day infected leaves to analyze gene expression of reference gene candidates *in planta*, because gene expressed in leaves at 10 and 15 days after infection showed similar CT values, and the amount of pathogen in these infected leaves was much more than at 5 day old infected leaves.

### Statistical analysis of real-time RT-PCR data by geNorm

An initial study of expression of all the genes selected as reference gene candidates (*EF1*, *EF2*, *EF3*, *UBC*, *UBCE2* and *UBQ*), was performed at four different growth stages (urediniospores, germinated spores, and leaves at 10 and 15 days after infection).

From the analysis of spores and germ tubes, genes *UBC* and *EF2* were estimated to have the lowest M value of 0.114 and hence the highest stability, while *UBCE2* gave the highest M value (lowest stability) (Table 3). Analysis of the infected leaves (10 and 15 d) showed similar results wherein the genes *UBC* and *EF2* had the lowest M value (the highest stability) of 0.174 (Table 4). As shown by a V value of 0.098 obtained from spores and germinated spores (Table 3) and 0.102 in infected leaves (Table 4), the use of the two most stably expressed genes, *UBC* and *EF2*, as reference genes are sufficient for reliable data normalization in this expression analysis in *P. helianthi*.

**Table 3:** Expression stability measures (M) calculated by geNorm for six genes analyzed in urediospores and germinated spores

Ranking order	Gene	Average expression stability M (^a^)	Pairwise variations V
1	*UBC*	0.114	
1	*EF2*	0.114	0.098
2	*EF3*	0.237	0.122
3	*EF1*	0.373	0.142
4	*UBQ*	0.517	0.173
5	*UBCE2*	0.706	

^a^Lower M values indicate higher expression stability

**Table 4:** Expression stability measures (M) calculated by geNorm for six genes analyzed in infected leaves

Ranking order	Gene	Average expression stability M	Pairwise variations V
1	*UBC*	0.174	
1	*EF2*	0.174	0.102
2	*UBCE2*	0.263	0.158
3	*EF1*	0.454	0.106
4	*EF3*	0.507	0.118
5	*UBQ*	0.588	

### Statistical analysis of real-time RT-PCR data by BestKeeper

The descriptive statistics of the six genes based on expression at different growth stage are presented in Tables 5 and 6. BestKeeper expresses the CT range of each individual gene as the extreme values of CT towards the geometric mean CT, and gives their standard deviations, hence providing an evaluation of the expression stability of each reference gene candidate (Tables 5 and 6 (min, max) [x-fold] and SD [± x-fold], respectively).

**Table 5:** BestKeeper analysis of reference gene expression stability in urediniospores and germinated spores

Factors (^a^)	Gene					
	*EF1*	*EF2*	*EF3*	*UBC*	*UBCE2*	*UBQ*
Geo mean [Ct]	23.60	18.87	21.38	21.92	25.68	25.12
Ar mean [Ct]	23.61	18.88	21.39	21.93	25.72	25.14
min [Ct]	22.76	18.17	20.92	21.22	24.17	23.72
max [Ct]	24.99	19.44	22.15	22.55	27.31	26.37
SD [Ct]	0.77	0.54	0.45	0.61	1.52	0.83
CV[% Ct]	3.26	2.84	2.11	2.80	5.92	3.31
Coeff. of corr.[r]	0.92	0.99	0.93	1.00	0.99	0.88
Coeff. of det.[r^2^]	0.84	0.99	0.87	0.99	0.97	0.77

^a^Geo mean [Ct]: Geometric mean of Ct value; Ar mean [Ct]: Arithmetic mean of Ct value; SD [Ct]: Standard deviation of Ct value; CV [%Ct]: Coefficient of variation of Ct value; Coeff. of corr. [r]: Coefficient of correlation r; Coeff. of det. [r^2^]: Coefficient of determination r^2^ between the reference gene and BestKeeper. The same as below

**Table 6:** BestKeeper analysis of reference gene expression stability in infected leaves

Factors	Gene					
	*EF1*	*EF2*	*EF3*	*UBC*	*UBCE2*	*UBQ*
Geo mean [Ct]	24.98	22.32	24.43	24.80	25.10	25.83
Ar mean [Ct]	24.99	22.32	24.44	24.80	25.18	25.84
min [Ct]	24.41	21.99	23.94	24.59	24.79	25.18
max [Ct]	25.69	22.48	25.30	25.03	25.57	26.97
SD [Ct]	0.35	0.17	0.43	0.16	0.20	0.63
CV[% Ct]	1.40	0.74	1.76	0.63	0.81	2.42
Coeff. of corr.[r]	0.72	0.79	0.98	0.08	−0.56	0.89
Coeff. of det.[r^2^]	0.52	0.62	0.96	0.01	0.32	0.79

In spores and germinated spores, *EF3* with the lowest CV ± SD value of 2.11 ± 0.45, was identified as the most stable gene. *EF2* (2.84 ± 0.54) and *UBC* (2.80 ± 0.61) were ranked as the second and third stable reference genes for normalization (Table 5). These gene stabilities were ranked as *EF3*>*EF2*>*UBC*>*EF1*>*UBQ*>*UBCE2*. *UBCE2* with SD greater than 1 was considered unacceptable and should be excluded.

*In planta*, *UBC* with the lowest CV ± SD value of 0.63 ± 0.16, was identified as the most stable gene. *EF2* (0.74 ± 0.17) and *UBCE2* (0.81 ± 0.20) were ranked as the second and third stable reference genes for normalization (Table 6). These gene stabilities were ranked as *UBC*>*EF2*>*UBCE2*>*EF1*>*EF3*>*UBQ*.

### Statistical analysis of real-time RT-PCR data by NormFinder

Based on NormFinder analysis, the stability in spores and germ tubes was (from the most stable to the least stable) *UBC > EF2 > EF1 > EF3 > UBQ > UBCE2* (Table 7). *UBC* had the most stable expression pattern among the samples. NormFinder also determined the stability value for the best combination of two genes, which is 0.002 for *UBC* and *EF2*.

**Table 7:** NormFinder analysis of reference gene expression stability in urediospores and germinated spores

Ranking order	Gene	M values
1	*UBC*	0.001
2	*EF2*	0.004
3	*EF1*	0.012
4	*EF3*	0.012
5	*UBQ*	0.021
6	*UBCE2*	0.030
Best combination of two genes	*UBC* + *EF2*	0.002

The stability in infected leaves was (from the most stable to the least stable) *UBC > EF2 > EF1 > EF3 > UBC > UBCE2 > UBQ*. The value for the best combination of two genes in *EF1* and *EF2*, was 0.005 (Table 8).

**Table 8:** NormFinder analysis of reference gene expression stability in infected leaves

Ranking order	Gene	M values
1	*EF2*	0.004
2	*EF1*	0.007
3	*EF3*	0.009
4	*UBC*	0.010
5	*UBCE2*	0.015
6	*UBQ*	0.021
Best combination of two genes	*EF1* + *EF2*	0.005

### Comprehensive stability analysis of reference genes

We determined the expression stability of six selected reference genes in *P. helianthi*. Eleven reference genes were validated in *P. helianthi* infection growth stages. In spores and germinated spores, the results obtained using geNorm, and NormFinder indicated that the two reference genes *UBC* and *EF2* were stable in *P. helianthi*. The results from BestKeeper showed *EF3* was the most stable followed by *EF2* and *UBC*. In the three programs combined, clarified how the *UBQ* and *UBCE2* genes were ranked as the least stable transcripts compared to the others. Moreover, the analysis of all the reference genes together indicated that *UBC* and *EF2* genes were the most stable (Tables 9 and 10). In infected leaves, geNorm and BestKeeper gave the similar results, indicating that both *UBC* and *EF2* ranked as the most stable transcripts. Also, NormFinder showed that *EF2* was the most stable.

**Table 9:** Expression stability ranking of the six candidate reference genes expressed in urediospores and germinated spores

Method	Ranking order (better-good-average)					
	1	2	3	4	5	6
geNorm	*UBC*	*EF2*	*EF3*	*EF1*	*UBQ*	*UBCE2*
NormFinder	*UBC*	*EF2*	*EF1*	*EF3*	*UBQ*	*UBCE2*
BestKeeper	*EF3*	*EF2*	*UBC*	*EF1*	*UBQ*	*UBCE2*
Comprehensive Ranking	*UBC*	*EF2*	*EF3*	*EF1*	*UBQ*	*UBCE2*

**Table 10:** Expression stability ranking of the six candidate reference genes expressed in infected leaves

Method	Ranking order (better-good-average)					
	1	2	3	4	5	6
geNorm	*UBC*	*EF2*	*UBCE2*	*EF1*	*EF3*	*UBQ*
NormFinder	*EF2*	*EF1*	*EF3*	*UBC*	*UBCE2*	*UBQ*
BestKeeper	*UBC*	*EF2*	*UBCE2*	*EF1*	*EF3*	*UBQ*
Comprehensive Ranking	*UBC*	*EF2*	*UBCE2*	*EF1*	*EF3*	*UBQ*

Comprehensive analysis of both results from spores and infected leaves with these three programs indicated *UBC* and *EF2* were the most stable genes.

## Discussion

Little was known about validation of a set of reference genes to be used in gene expression experiments in *P. helianthi*. The specificity of internal reference genes should be examined when studying *P. helianthi* sunflower interactions. Therefore, in addition to the detection of primers specificity for the rust cDNA amplification, amplification of sunflower cDNA with these primers was also tested. The *ACTB* gene marker was discarded for real time PCR to study rust gene expression in sunflower, because it could give an amplicon in the non-infected leaves. However, the *ACTB* primers still could be used for real time PCR research to study only rust gene expression in vitro.

When we adopted the analysis software to screen suitable reference genes of certain species of fungi, sample selection influenced test result. Therefore, while analyzing, samples from different growth stages and conditions should be considered. Since *P. helianthi* cannot be reproduced in vitro, we chose samples of pathogen and inoculated sunflower leaves at different growth stages for reference gene selection.

Using the comparison of CT methods, we analyzed the gene expression of each housekeeping gene in urediniospore, germinated spores and 5, 10 and 15d infected leaves. The total amounts of cDNA in all samples were the same, deriving from 2 μg total RNA, but the amount of rust pathogen in different samples differed greatly. Five day post-inoculation was at its primary infection stage, hence the leaf sample only contained a small amount of hyphae and haustoria, with no visible symptoms. Whereas leaf samples of 10 d post-inoculation had more of pathogen mycelia with obvious symptoms and in 15 d post-inoculation the fungus produced a large number of uredia, covering the whole leaf, indicating dramatically increased severities compared with 5 d infected leaf samples. Gene *RPS24* was not suitable as candidate, for it showed higher expression level in infected leaves than in spores. Expression levels of gene *RPS5*, *TUBA*, *TUBB* were very low in all samples, hence, these three genes were not suitable as candidate internal reference genes in *P. helianthi* too. However, β-tubulin was recommended as the best option for normalization in highly virulent plant pathogen *Lasiodiplodia theobromae* [22].

Biomass of pathogenic fungi varied considerably during their infection processes *in planta*, which raises the need for an adequate method for further normalization of the proportion of fungal cDNA in the total plant and fungus cDNA pool [23]. Detecting the expression of different genes in post-inoculated leaves, demonstrated that with the increase of the pathogen biomass in the leaves, the gene expression of *EF1*, *UBQ*, *UBC*, *EF3*, *UBCE2*, *EF2* increased significantly. The expression of these six housekeeping genes was consistent with the macro change rule of sunflower rust, and basically met the condition as internal reference gene. Expression of these genes at 10 and 15 d infected leaves were similar. Therefore, 10 and 15 d infected leaves were chosen for gene expression analysis under the condition of interaction (*in planta*). Urediospores and germinated spores were chosen for analysis in vitro. Vieira et al. [23] studied the expression of seven reference genes, namely glyceraldehyde-3-phosphate dehydrogenase (*GADPH*), *EF-1*, β-tubulin, cytochrome c oxidase subunit III (*Cyt III*), cytochrome b (*Cyt b*), *Hv00099*, and 40S ribosomal protein (*40S_Rib*) in *Hemileia vastatrix*, the causal agent of coffee leaf rust, in vitro (germinated urediniospores and appressoria) and *in planta* (post-penetration fungal growth phases). Gene stability was assessed using geNorm and NormFinder tools. *Cyt b*, *40S_Rib*, and *Hv00099* were the most stable genes in vitro, while *40S_Rib*, *GADPH*, and *Cyt III* were the most stable *in planta*. For the combined datasets (in vitro and *in planta*), *40S_Rib*, *GADPH*, and *Hv00099* were selected as the most stable.

Dankai et al. [24] reported that the actin gene was the most stably expressed among selected four housekeeping genes, namely β-actin, glyceraldehyde-3-phosphate dehydrogenase, β-tubulin and 18S rRNA, and was recommended for use as the endogenous control for gene expression analysis of all growth forms in *Talaromyces marneffei* by qRT-PCR under normal and stress conditions. Their results showed that it still could not meet the requirement for qRT-PCR standardization analysis (M value >1.5).

Interestingly, the actin transcript, which has been extensively used as internal contol in qRT-PCR, is not the best choice after a wide range of reference gene selection. Kummasook et al. [25] reported during the mycelium to yeast phase transition of *Penicillium marneffei* the actin transcripts were initially upregulated soon after shifting the incubation temperature from 25 °C to 37 °C, but subsequently decreased slightly and did not change during further growth or under stress conditions. This showed that actin expression was not stable under different conditions. Tao et al. [26] reported that F-actin capping protein alpha subunit (*FacpA*) and vacuolar protein sorting protein (*DigA*) were the optimum pairs of reference genes which were more stable than β-actin at all dimorphic phase transition stages and in two different strains of *P. marneffei*, and were recommended for use as the endogenous control for gene expression analysis in this pathogenic fungus by qRT-PCR.

Ideal reference genes should be expressed at constant levels in all samples under various experimental conditions [27]. With the improvement of qRT-PCR technology, more studies for validation of suitable reference genes have been carried out. Many studies prefer to use several reference genes, and use the geometric mean of the relative expression values from the housekeeping genes for normalization, which can avoid misinterpretation of gene expression data [28].

The selection of internal genes for qRT-PCR based on large-scale transcriptome sequencing data is a relatively new and effective strategy [29–31]. A new set of internal genes was selected and identified by Cankorur-Cetinkaya et al. [29] who employed this strategy, and these genes have been successfully applied in qRT-PCR in filamentous fungi [32], plants [30] and animals [31, 33].

Several mathematical approaches (computing algorithms) deliver suitable reference genes with the lowest variation and with high stability across biological samples. The four most commonly used approaches are NormFinder, geNorm, BestKeeper and the comparative delta Ct. Neither of these approaches can provide entirely satisfactory solution for choosing which reference gene to use and how to identify it. This may also affect the interpretation of qRT-PCR results. In order to ensure the reliability of gene expression analyses, we conducted comprehensive assessment of reference genes based on the results derived from the algorithms used.

The current study is the first systematic evaluation of the performance of potential reference genes as normalizers in *P. helianthi* expression studies. Eleven genes were selected from RNA-seq data for gene expression stability analysis. On the basis of the resulted rankings from the three algorithms (geNorm, Bestkeeper and NormFinder), we obtained the suitable combination (*UBC* and *EF2*) of reference genes of *P. helianthi*. Our results provide a useful reference for studying gene expression in the interaction between other rust pathogens and their hosts.

## Conclusions

This is the first study to conduct a systematic exploration of *P. helianthi* to validate candidate reference genes for qRT-PCR normalization in urediniospores, germ tubes and infected leaves of different developmental stages. Eleven housekeeping genes were assessed. *UBC* and *EF2* were identified as optimum internal control genes at different developmental stages (pure pathogen or interaction with the host) with three computer algorithms geNorm, BestKeeper and NormFinder. These results present useful information for reliable qRT-PCR data normalization in *P. helianthi* gene expression studies.

## Abbreviations

*40S_Rib*: 40S ribosomal protein
*ACTB*: β-actin
*Cyt b*: Cytochrome b
*Cyt III*: Cytochrome c oxidase subunit III
*DigA*: Vacuolar protein sorting protein
*EF1*: Elongation factor 1
*EF2*: Elongation factor 2
*EF3*: Elongation factor 3
*FacpA*: F-actin capping protein alpha subunit
*GADPH*: Glyceraldehyde-3-phosphate dehydrogenase
qRT-PCR: Quantitative real-time polymerase chain reaction
*RPS24*: Ribosomal protein S24
*RPS5*: Ribosomal protein S5
*TUBA*: α-tubulin
*TUBB*: β-tubulin
*UBC*: Ubiquitin-conjugating enzyme
*UBCE2*: E2 ubiquitin-conjugating enzyme
*UBQ*: Polyubiquitin

## References

[1] Galli V, Borowski JM, Perin EC, Messias RS, Labonde J, Pereira IS, Silva SD, Rombaldi CV (2015). Validation of reference genes for accurate normalization of gene expression for real time-quantitative PCR in strawberry fruits using different cultivars and osmotic stresses. Gene.

[2] Machado RD, Christoff AP, Loss-Morais G, Margis-Pinheiro M, Margis R, Körbes AP (2015). Comprehensive selection of reference genes for quantitative gene expression analysis during seed development in *Brassica napus*. Plant Cell Rep.

[3] Huggett J, Dheda K, Bustin S, Zumla A (2005). Real-time RT-PCR normalisation; strategies and considerations. Genes Immun.

[4] Radonic A, Thulke S, Mackay IM, Landt O, Siegert W, Nitsche A (2004). Guideline to reference gene selection for quantitative real-time PCR. Biochem Biophys Res Commun.

[5] López MM, Bertolini E, Olmos A, Caruso P, Gorris MT, Llop P, Penyalver R, Cambra M (2003). Innovative tools for detection of plant pathogenic viruses and bacteria. Int Microbiol.

[6] Schneider S, Widmer F, Jacot K, Koelliker R, Enkerli J (2012). Spatial distribution of Metarhizium clade 1 in agricultural landscapes with arable land and different semi-natural habitats. Appl Soil Ecol.

[7] Iakovlev A, Olson A, Elfstrand M, Stenlid J (2004). Differential gene expression during interactions between *Heterobasidion annosum* and *Physisporinus sanguinolentus*. FEMS Microbiol Lett.

[8] Karlsson M, Stenlid J, Olson A (2007). Two hydrophobin genes from the conifer pathogen *Heterobasidion annosum* are expressed in aerial hyphae. Mycologia.

[9] Yakovlev IA, Hietala AM, Steffenrem A, Solheim H, Fossdal CG (2008). Identification and analysis of differentially expressed *Heterobasidion parviporum* genes during natural colonization of Norway spruce stems. Fungal Genet Biol.

[10] Scholtz JJ, Visser B (2013). Reference gene selection for qRT-PCR gene expression analysis of rust-infected wheat. Physiol Mol Plant Pathol.

[11] Kim HK, Yun SH (2011). Evaluation of potential reference genes for quantitative RT-PCR analysis in *Fusarium graminearum* under different culture conditions. Plant Pathol J.

[12] Bohle K, Jungebloud A, Gocke Y, Dalpiaz A, Cordes C, Horn H, Hempel DC (2007). Selection of reference genes for normalisation of specific gene quantification data of *Aspergillus niger*. J Biotechnol.

[13] Che OS, Bentley MA, Morieri G, Preston GM, Gurr SJ (2016). Validation of reference genes for robust qRT-PCR gene expression analysis in the rice blast fungus *Magnaporthe oryzae*. PLoS One.

[14] Guenin S, Mauriat M, Pelloux J, Van Wuytswinkel O, Bellini C, Gutierrez L (2009). Normalization of qRT-PCR data: the necessity of adopting a systematic, experimental conditions-specific, validation of references. J Exp Bot.

[15] Gutierrez L, Mauriat M, Guenin S, Pelloux J, Lefebvre JF, Louvet R, Rusterucci C, Moritz T, Guerineau F, Bellini C, Van Wuytswinkel O (2008). The lack of a systematic validation of reference genes: a serious pitfall undervalued in reverse transcription-polymerase chain reaction (RT-PCR) analysis in plants. Plant Biotechnol J.

[16] Schmittgen TD, Livak KJ (2008). Analyzing real-time PCR data by the comparative CT method. Nat Protoc.

[17] Thellin O, Zorzi W, Lakaye B, De Borman B, Coumans B, Hennen G, Grisar T, Igout A, Heinen E (1999). Housekeeping genes as internal standards: use and limits. J Biotechnol.

[18] Pfaffl MW, Horgan GW, Dempfle L (2002). Relative expression software tool (REST) for group-wise comparison and statistical analysis of relative expression results in real-time PCR. Nucleic Acids Res.

[19] Vandesompele J, De Preter K, Pattyn F, Poppe B, Van Roy N, De Paepe A, Speleman F (2002). Accurate normalization of real-time quantitative RT-PCR data by geometric averaging of multiple internal control genes. Genome Biol.

[20] Andersen CL, Jensen JL, Orntoft TF (2004). Normalization of real-time quantitative reverse transcription-PCR data: a model-based variance estimation approach to identify genes suited for normalization, applied to bladder and colon cancer data sets. Cancer Res.

[21] Pfaffl MW, Tichopad A, Prgomet C, Neuvians TP (2004). Determination of stable housekeeping genes, differentially regulated target genes and sample integrity: BestKeeper–excel-based tool using pair-wise correlations. Biotechnol Lett.

[22] Paolinelli-Alfonso M, Galindo-Sánchez CE, Hernandez-Martinez R (2016). Quantitative real-time PCR normalization for gene expression studies in the plant pathogenic fungi *Lasiodiplodia theobromae*. J Microbiol Methods.

[23] Vieira A, Talhinhas P, Loureiro A, Duplessis S, Fernandez D, Silva Mdo C, Paulo OS, Azinheira HG (2011). Validation of RT-qPCR reference genes for in planta expression studies in *Hemileia vastatrix*, the causal agent of coffee leaf rust. Fungal Biol.

[24] Dankai W, Pongpom M, Vanittanakom N (2015). Validation of reference genes for real-time quantitative RT-PCR studies in *Talaromyces marneffei*. J Microbiol Methods.

[25] Kummasook A, Tzarphmaag A, Thirach S, Pongpom M, Cooper CR, Vanittanakom N (2011). *Penicillium marneffei* actin expression during phase transition, oxidative stress, and macrophage infection. Mol Biol Rep.

[26] Tao Y, Lu S, Wang C, Wang J, Kang X, Lan X, Liang X, Liang Y, He Z (2016). Selection and identification of reference genes for qRT-PCR in *Penicillium marneffei* based on RNA-Seq. Genomics Appl Biol.

[27] Banda M, Bommineni A, Thomas RA, Luckinbill LS, Tucker JD (2008). Evaluation and validation of housekeeping genes in response to ionizing radiation and chemical exposure for normalizing RNA expression in real-time PCR. Mutat Res.

[28] Nailis H, Coenye T, Van Nieuwerburgh F, Deforce D, Nelis HJ (2006). Development and evaluation of different normalization strategies for gene expression studies in *Candida albicans* biofilms by real-time PCR. BMC Mol Biol.

[29] Cankorur-Ceinkaya A, Dereli E, Eraslan S, Karabekmez E, Dikicioglu D, Kirdar B (2012). A novel strategy for selection and validation of reference genes in dynamic multidimensional experimental design in yeast. PLoS One.

[30] Chang E, Shi S, Liu J, Cheng T, Xue L, Yang X, Yang W, Lan Q, Jiang Z (2012). Selection of reference genes for quantitative gene expression studies in *Platycladus orientalis* (Cupressaceae) using real-time PCR. PLoS One.

[31] Zhan C, Zhang Y, Ma J, Wang L, Jiang W, Shi Y, Wang Q (2014). Identification of reference genes for qRT-PCR in human lung squamous-cell carcinoma by RNA-Seq. Acta Biochim Biophys Sin.

[32] Vieira A, Cabral A, Fino J, Azinheira HG, Loureiro A, Talhinhas P, Pires AS, Varzea V, Moncada P, Oliveira H, Silva MC, Paulo OS, Batista D (2016). Comparative validation of conventional and RNA-Seq data-derived reference genes for qRT-PCR expression studies of *Colletotrichum kahawae*. PLoS One.

[33] Hu Y, Xie S, Yao J (2016). Identification of novel reference genes suitable for qRT-PCR normalization with respect to the zebrafish developmental stage. PLoS One.

